# Glutathione Depletion in Acute Respiratory Distress Syndrome: Mechanistic Significance and Potential Association with Ventilatory Support

**DOI:** 10.3390/ijms27198592

**Published:** 2026-09-25

**Authors:** Brandon Tran, Elaine Nguyen, Srinivasan Karthikeyan, Shady Ibrahim, Frederick Timothy Guilford, Vishwanath Venketaraman

**Affiliations:** 1 College of Osteopathic Medicine of the Pacific, Western University of Health Sciences, 309 E. Second St., Pomona, CA 91766, USA; brandon.tran@westernu.edu (B.T.); elaine.nguyen1@westernu.edu (E.N.); seenu0610@gmail.com (S.K.); shady.ibrahim@westernu.edu (S.I.); 2 Your Energy System, LLC, 555 Bryant St. #305, Palo Alto, CA 94301, USA; drg@readisorb.com

**Keywords:** GSH, mechanical ventilation, ARDS, COVID-19, thrombo-inflammation, ROS, SARS-CoV2

## Abstract

Acute Respiratory Distress Syndrome (ARDS) is a severe and life-threatening form of acute lung injury with high mortality. It affects approximately 150,000–190,000 patients annually in the United States, and a substantial proportion of patients require invasive mechanical ventilation for respiratory support. The progression of ARDS involves thrombo-inflammation, the disruption of the alveolar–capillary barrier, and oxidative injury within pulmonary tissues. Across diverse ARDS etiologies such as sepsis, aspiration, trauma, and COVID-19, the excessive production of reactive oxygen species (ROS) is a central mediator of lung injury. Central to the neutralization of oxidative stress is glutathione (GSH), a key intracellular antioxidant responsible for neutralizing ROS. Decreased levels of GSH have been associated with increased cytokine levels and thrombosis, but the full extent of GSH’s role in these processes remains unclear. This review provides an overview of the role of glutathione in the pathophysiological sequelae of ARDS, highlights its relevance in COVID-19-related lung injury, and explores its potential as a clinical marker to predict the need for mechanical ventilation.

## 1. Introduction

Acute Respiratory Distress Syndrome (ARDS) is defined as a severe acute respiratory failure with rapid-onset inflammation and damage to the lungs, resulting in capillary endothelial and alveolar injury. It normally occurs with signs of lung infiltrations, severe hypoxemia, and non-cardiogenic edema. Historically, ARDS has been defined according to the 2012 Berlin definition, which is categorized into [1]:

Timing: Acute onset within 7 days of a known clinical insult or worsening respiratory symptoms.

Radiology: Bilateral opacities not explained by lung nodules, effusions, mass, or lung collapse.

Edema: Respiratory failure not explained by cardiac failure or fluid overload.

Degree of ARDS

Mild: PaO_2_/FiO_2_ between 200 and 300 mm Hg.

Moderate: PaO_2_/FiO_2_ between 100 and 200 mm Hg.

Severe: PaO_2_/FiO_2_ ≤ 100 mm Hg.

More recently, the Global Definition of ARDS has expanded the diagnostic framework to improve the recognition of ARDS across a broader range of clinical settings and respiratory support modalities. The Global Definition of ARDS retains the main features of acute hypoxemic respiratory failure and pulmonary abnormalities while expanding the criteria to include patients receiving noninvasive respiratory support. This definition also permits the use of oxygen saturation (SpO_2_/FiO_2_) as an alternative measure of oxygenation when arterial blood gas measurements are unavailable and incorporates lung ultrasound as an acceptable imaging modality [2]. While the Global Definition represents a more contemporary framework for identifying ARDS, the Berlin definition remains important for interpreting the existing literature, as many of the clinical studies discussed in this review were conducted using Berlin criteria.

Despite advances in medicine, ARDS is associated with a high mortality rate. The incidence of ARDS in the United States ranges from 64.2 to 78.9 cases/100,000 person-years. Around 10 to 15% of patients admitted to the ICU and up to 23% of mechanically ventilated patients meet the criteria for ARDS [1]. In another study, done in Europe, from 2002 to 2022, the current reports of ARDS showed a wide range of incidence (7.2–78.9/100,000 population/year) and hospital mortality (32–51%), with the effects of the COVID-19 pandemic contributing to the statistics. Mechanically ventilated ARDS incidence ranged from 2.96 to 20.14/100,000 population-years, peaking in 2021. Mortality ranged between 38.0 and 55.0%, showing a declining trend [3].

The management of ARDS is primarily supportive, mainly with mechanical ventilation. Mechanical ventilation is necessary when the patient develops severe hypoxemia that is noncompliant with supplemental oxygen. The purpose of mechanical ventilation in ARDS is to improve oxygenation, reduce respiratory effort and ultimately prevent further lung injury. The need for ventilation is based on PaO_2_/FiO_2_ levels as stated above. Studies have shown that the protocol for ventilation is: low tidal volume (TV) from 4 to 8 mL/kg of ideal body weight (IBW), respiratory rate (RR) up to 35 bpm, SpO_2_ 88% to 95%, plateau pressure (Pp) less than 30 cm H_2_O, pH goal 7.30 to 7.45, and inspiratory-to-expiratory time ratio less than 1. High positive end expiratory pressure (PEEP) has also been shown to reduce mortality rates [1,4].

Mechanical ventilation is an important supportive therapy in patients with ARDS who develop severe hypoxemic respiratory failure. However, inappropriate mechanical ventilation can exacerbate pulmonary injury through ventilator-induced lung injury, making lung-protective ventilation essential in limiting further alveolar and endothelial damage. Recent experimental evidence suggests that injurious mechanical stress may also amplify oxidative lung injury through NOX2-derived ROS, which can contribute to pulmonary endothelial dysfunction and the disruption of endothelial junction integrity [5]. ARDS is characterized by increased lung inflammation and the accumulation of ROS, which can induce endothelial injury in lung tissue. The imbalance between ROS and antioxidants, particularly GSH, may further contribute to the severity of ARDS and hypoxemia. GSH depletion appears to have an inverse correlation with the severity of illness [6]. Therefore, targeting GSH levels may be therapeutic in reducing the morbidity and mortality associated with ARDS (Table 1).

Evidence that GSH diminishment accompanies ARDS predates the COVID-19 pandemic. Rahman and MacNee summarized the measurements of the reduced form of GSH in epithelial lining fluid obtained by bronchoalveolar lavage and reported concentrations of 31.5 ± 8.4 μM in patients with ARDS compared with 651 ± 103.1 μM in controls. This marked difference demonstrates that the depletion of the antioxidant capacity of the pulmonary epithelial lining fluid is a characteristic of established ARDS. Because epithelial lining fluid is positioned at the air–lung interface, these findings provide evidence of a local pulmonary redox disturbance rather than solely a change in circulating antioxidant status. Rahman and MacNee further proposed that a low epithelial lining fluid GSH concentration may amplify inflammatory responses and potentiate lung injury (Table 1). These observations provide an important pathophysiological foundation for subsequent studies evaluating glutathione in acute respiratory failure [7].

Table 1 shows the proposed pathophysiological framework linking GSH depletion to oxidative stress, endothelial dysfunction, thrombo-inflammation, and progressive respiratory failure in ARDS. ARDS resulting from diverse etiologies, including SARS-CoV-2 infection, sepsis, trauma, and aspiration, shares common mechanisms characterized by excessive ROS production, the depletion of the reduced form of GSH, endothelial dysfunction, inflammatory activation, coagulation abnormalities, and impaired pulmonary gas exchange. This table summarizes the mechanistic pathways and representative biomarkers discussed throughout this review. Current evidence supports an association between GSH depletion and increasing disease severity; however, whether GSH independently predicts progression to mechanical ventilation remains to be established in prospective clinical studies.

Several recent articles have reviewed the roles of oxidative stress, inflammation, and cell death in ARDS. However, these reviews primarily provide a broad overview of these mechanisms and do not specifically examine the relationship between GSH depletion, pulmonary thrombo-inflammation, and progressive respiratory failure. For instance, Sergazy et al. discussed antioxidant and cytoprotective mechanisms in respiratory diseases, as well as the molecular interactions among ferroptosis, pyroptosis, and other inflammatory pathways, without specifically addressing their relationship to GSH depletion [8]. Although previous studies have identified reduced GSH levels in ARDS and severe COVID-19, the relationship between GSH depletion, oxidative stress, coagulation abnormalities, and progression to respiratory failure has not been comprehensively evaluated. The utility of GSH depletion as a predictor of the need for mechanical ventilation remains unvalidated and requires further prospective investigation. Therefore, this review aims to focus on the mechanistic role of GSH depletion in the pathophysiology of ARDS, with particular emphasis on its potential relationship with thrombo-inflammation and its possible utility as a biomarker associated with disease severity and the need for mechanical ventilation.

### GSH and Its Antioxidant Function

GSH is an intracellular tripeptide made up of gamma-Glutamyl-cysteinyl glycine. It is found in the body in its reduced state (GSH) and is involved in antioxidant protection against ROS and RNI. The balance between its reduced and oxidized (GSSG) forms (redox cycle) is suggestive of a cell’s overall health (Figure 1). GSH is secreted by the epithelial cells lining the pneumocytes to protect the lungs from damage [9]. The mechanism of how GSH neutralizes oxygen species involves donating a hydrogen atom to ROS, in the process becoming oxidized to GSSG by glutathione peroxidase and GSH-s-transferase. To complete the cycle, GSH reductase converts GSSG back into GSH through the salvage pathway with the use of NADPH, thus replenishing cellular GSH [10]. GSH can also replenish other antioxidants, such as vitamin E, after its detoxification of lipid peroxyl radicals [9]. This cycle maintains the oxidative stress balance in the cells, which results in the protection of the body from oxidative damage (Figure 1). A recent single-cell transcriptomic analysis of human ARDS lung tissue provides further evidence that this antioxidant system is disrupted in ARDS, demonstrating reduced GSH peroxidase 3 expression in alveolar macrophages that was associated with enhanced IL-17-related inflammatory signaling [11]. These findings provide evidence linking impaired GSH-dependent antioxidant defenses with inflammatory activation in the ARDS lung.

In addition to detoxifying peroxides, the GSH/GSSG system participates in the regulation of redox-sensitive inflammatory signaling. A highly reduced intracellular environment limits the accumulation of oxidized proteins and preserves normal cellular signaling, whereas the depletion of GSH and accumulation of GSSG alter cellular redox homeostasis. Oxidative stress can activate NF-κB, AP-1, JNK, and p38 signaling pathways, thereby increasing the expression of inflammatory cytokines, chemokines, and endothelial adhesion molecules. ROS can also induce lipid, protein, and DNA oxidation; mitochondrial dysfunction; and apoptotic signaling when endogenous antioxidant defenses are insufficient. In respiratory viral infections, these redox disturbances are linked to inflammatory signaling, tissue injury, and cell death. Thus, a GSH decrease may contribute to an intracellular environment that permits the amplification of pulmonary inflammation; however, the magnitude and direction of these effects depend on the cell type, stimulus, and degree of redox disturbance [9,12,13].

As shown in Figure 1, GSH in its reduced form neutralizes ROS through the enzyme glutathione peroxidase by catalyzing the reduction of H_2_O_2_ and converting GSH to its oxidized form (GSSG). GSH reductase then regenerates GSH from GSSG using NADPH, maintaining cellular redox balance.

## 2. GSH Depletion and Its Role in Thrombo-Inflammation and Respiratory Failure in COVID-19

Building on this redox background, ARDS cases arising from different etiologies like pneumonia, trauma, sepsis, and viral infections share a final common pathway characterized by oxidative stress, inflammatory injury, and alveolar–capillary barrier disruption (Table 1). COVID-19-associated ARDS represents a well-characterized example of this process, with particularly pronounced thrombo-inflammatory and microvascular features, and is therefore used here to illustrate these broader pathophysiological mechanisms.

GSH depletion, frequently observed in patients with COVID-19, has emerged as an important contributor to thrombotic complications that exacerbate pulmonary dysfunction and respiratory distress (Figure 2). Clinically, COVID-19 patients commonly present with prolonged prothrombin time and elevated D-dimer levels at hospital admission, reflecting the activation of coagulation pathways and ongoing fibrin turnover [14]. SARS-CoV-2 further promotes pulmonary vascular thrombosis by potentiating all three components of Virchow’s triad—hypercoagulability, blood flow stasis, and endothelial injury—thereby increasing thrombotic risk and contributing to reduced static lung compliance [15]. The combined presence of elevated D-dimer levels and decreased static lung compliance has been consistently associated with greater disease severity and adverse clinical outcomes (Figure 2). In a prospective study of 102 prolonged hospitalized COVID-19 patients, 25.5% were found to have asymptomatic deep vein thrombosis (DVT). Mechanically ventilated ICU survivors with respiratory failure had the highest rate of asymptomatic DVT at 60%, compared with about 21–22% in non-ICU patients. Notably, patients exhibiting both abnormalities demonstrate significantly higher mortality, highlighting the pathological link between thrombosis, parenchymal lung injury, and respiratory failure in COVID-19 [16].

As previously mentioned, oxidative stress is an important component of ARDS and may represent a common downstream pathway through which diverse insults amplify pulmonary injury. Excess ROS generated by sources such as NADPH oxidases and mitochondria can activate NF-κB and MAPK signaling and promote lipid and protein oxidation. In the lung, this can injure epithelial and endothelial cells, disrupt the alveolar–capillary barrier, increase vascular permeability, and worsen pulmonary edema and gas exchange [17].

To link GSH with thrombo-inflammatory events, GSH overall detoxifies reactive species and lipid peroxides. Sustained oxidative stress consumes GSH, shifting cells toward an oxidized state and increasing susceptibility to further injury. GSH depletion may therefore contribute to the progression of acute lung injury. Thrombo-inflammatory injury is thus possible in terms of endothelial dysfunction and barrier disruption. Increased ROS can amplify inflammatory signaling, cytokine production, and cellular injury while promoting programmed cell death pathways [18,19].

SARS-CoV-2 infection is associated with the disruption of cellular redox homeostasis, and excessive oxidant production has been proposed to contribute to pulmonary dysfunction, inflammatory amplification, coagulation abnormalities, and hypoxemic respiratory failure. Oxidative stress may reinforce inflammatory signaling and tissue injury, while impaired antioxidant defenses reduce the capacity of pulmonary and vascular cells to terminate these responses.

As shown in Figure 2, SARS-CoV-2 infection promotes thrombosis through the activation of all three components of Virchow’s triad: endothelial injury, blood flow stasis, and hypercoagulability. By inducing a cytokine storm, oxidative stress, and the need for hospitalization, this leads to a thrombo-inflammatory state that significantly increases the risk of thrombotic complications in patients with COVID-19. Alveolar endothelial cell injury can further increase hypercoagulation and blood stasis by reducing nitric oxide bioavailability, promoting vasoconstriction, and increasing the release of procoagulant factors.

### 2.1. Virchow’s Triad and Hypercoagulability in COVID-19

SARS-CoV-2 infection is found to directly potentiate hypercoagulability by triggering a strong systemic inflammatory response called a “cytokine storm”, increasing procoagulant factors like tissue factor, fibrinogen, factor VIII, and von Willebrand factor (vWF) [20]. These inflammatory mediators promote procoagulation by inducing tissue factor expression on endothelial cells and macrophages, increasing platelet adhesion, and disrupting normal anticoagulant signaling. In addition, significantly elevated levels of IL-6, TNF-α, ICAM-1, and caspase-1 have been identified in lung tissues from COVID-19 patients compared with controls [21]. Although these molecules are not coagulation factors themselves, they indirectly drive coagulation by activating endothelial cells, platelets, and immune pathways, thereby promoting thrombus formation. Beyond increasing procoagulant signaling, SARS-CoV-2–associated inflammation also suppresses endogenous anticoagulant mechanisms (Figure 3). IL-6, a pleiotropic pro-inflammatory cytokine, plays a role in procoagulation by reducing the synthesis of antithrombotic proteins like antithrombin in both in vivo and in vitro studies [22]. IL-6 has been further demonstrated to significantly decrease the levels of free protein S, a critical cofactor of the protein C anticoagulant pathway, thereby impairing endogenous anticoagulant function and promoting a prothrombotic state [23]. These inflammatory and coagulation pathways are further amplified under conditions of oxidative stress, where the depletion of GSH exacerbates endothelial dysfunction and platelet activation [24].

As shown in Figure 3, after binding to ACE2 receptors on alveolar epithelial and endothelial cells, SARS-CoV-2 stimulates macrophages and other immune cells to release pro-inflammatory cytokines, initiating a cytokine storm. These cytokines diffuse into the pulmonary capillaries and activate procoagulation factors like tissue factor, fibrinogen, factor VIII, and vWF.

### 2.2. Blood Flow Stasis and Thrombotic Risk

Beyond hypercoagulability, SARS-CoV-2 infection also promotes blood flow stasis, as severe cases frequently require prolonged hospitalization, mechanical ventilation, and extended periods of immobility [25]. Prolonged immobility is a well-recognized risk factor for DVT, primarily due to the absence of regular skeletal muscle contraction, which reduces venous return and promotes venous blood pooling and stasis [26]. In addition, COVID-19-associated hypoxia further exacerbates impaired blood flow by altering vascular tone, increasing blood viscosity, and disrupting microvascular perfusion [26]. Microvascular thrombosis may further promote local stasis by obstructing capillary beds, particularly within the pulmonary circulation. Endothelial injury initiated by SARS-CoV-2-induced inflammation may further contribute to stasis through endothelial dysfunction and vasoconstriction, limiting normal blood flow regulation [27]. Low intracellular GSH amplifies oxidative stress within endothelial cells, impairing nitric oxide bioavailability, promoting endothelial dysfunction, and shifting the vascular environment toward vasoconstriction and pro-adhesive states that favor impaired microcirculatory flow and blood pooling. Consistent with these mechanisms, a high incidence of venous thrombotic events has been reported in patients hospitalized with severe COVID-19, further supporting the contribution of infection-associated immobility and blood flow stasis to thrombosis risk [28].

### 2.3. Oxidative Stress, NOX2 Activation, and Endothelial Injury

Endothelial cell injury represents a third major contributor to thrombosis in COVID-19 and is closely linked to oxidative stress. Serum from patients with severe COVID-19 has been shown to significantly increase ROS production and lipid peroxidation in human endothelial cells, directly indicating heightened oxidative stress and highlighting the importance of upstream inflammatory mechanisms [24]. COVID-19 patients requiring intensive care also had higher circulating levels of soluble Nox2-derived peptide (sNox2-dp) compared with non-ICU patients. Nox2 is a major enzymatic source of reactive oxygen species, and these findings provide evidence that oxidative stress rises with COVID-19 severity.

Within the cohort, patients who developed thrombotic complications showed higher Nox2 activation and elevated D-dimer levels compared with event-free patients, implicating Nox2-mediated oxidative stress in promoting thrombotic ischemic events. Interestingly, this study reported that sNox2-dp levels did not correlate directly with D-dimer concentrations.

NOX2 activation primarily reflects upstream inflammatory and endothelial processes, including reactive oxygen species generation, endothelial barrier disruption, nitric oxide depletion, and platelet hyperreactivity [29]. Oxidative stress promotes endothelial injury and creates a prothrombotic environment even before substantial fibrin deposition or breakdown occurs. In contrast, D-dimer reflects established fibrin clot formation, such as previous pulmonary embolisms, and subsequent fibrinolysis rather than the initiating inflammatory and oxidative events [30]. Complementing this distinction, recent work demonstrates that fibrin itself can actively drive oxidative stress by inducing NADPH oxidase. This activation generates reactive oxygen species and links upstream inflammatory and oxidative events to thrombotic complications and severe disease in COVID-19 [29]. These data suggest a positive feedback cycle in which oxidative stress predisposes patients to thrombus formation, and once formed, fibrin promotes further oxidative inflammation.

Mitochondria provide an additional source of ROS during severe pulmonary infection. Mitochondrial dysfunction can impair oxidative phosphorylation while increasing the leakage of electrons from the respiratory chain, promoting the formation of superoxide and hydrogen peroxide. These oxidants can activate inflammatory signaling in macrophages, neutrophils, and endothelial cells and may stimulate further ROS generation through NADPH oxidase-dependent pathways. This interaction between mitochondrial and enzymatic ROS sources provides a plausible feed-forward mechanism through which oxidative stress becomes sustained as antioxidant reserves decline [12,31,32].

Recent evidence further links mitochondrial redox dysfunction to ferroptotic lung injury in ARDS. SERPINE1, which encodes plasminogen activator inhibitor-1 (PAI-1), is involved in inflammatory and coagulation pathways and has been associated with ARDS severity. One recent study found increased levels of SEREPINE1 in ARDS patients, and this was correlated with disease severity. Using clinical samples, experimental ARDS models, and alveolar type II epithelial cells, investigators found that SERPINE1 promoted ferroptosis, an iron-dependent form of regulated cell death driven by lipid peroxidation. Mechanistically, SERPINE1 disrupted mitochondrial NAD+/NADH homeostasis and suppressed Sirt3 activity, resulting in mitochondrial dysfunction and enhanced ferroptotic signaling. SERPINE1 inhibition reduced lung injury and ferroptosis while restoring ferroptosis-inhibitory proteins, including SLC7A11 and GSH peroxidase 4 [33]. These findings provide additional evidence that mitochondrial redox imbalance can contribute to lipid peroxidation and ferroptotic epithelial injury in ARDS.

Additionally, excess ROS further damages endothelial membranes and proteins and impairs nitric oxide bioavailability, which is important in maintaining vascular tone, inhibiting platelet aggregation, and preserving endothelial barrier integrity. This impairment of NO bioavailability leads to increased vasoconstriction and enhanced platelet aggregation, promoting microvascular thrombosis and endothelial damage, particularly within pulmonary tissue. This can be seen in a study where it was found that COVID-19 patients had reduced levels of hemoglobin-bound nitric oxide (HbNO), a marker of vascular NO bioavailability, along with microscopy evidence of SARS-CoV-2-induced endothelial damage within pulmonary blood vessels [34]. In the setting of depleted GSH, antioxidant defenses are further compromised, allowing ROS to accumulate and worsen endothelial dysfunction. Together, this ROS accumulation and impaired antioxidant defenses establish a self-perpetuating cycle of endothelial injury that plays a role in thrombosis and pulmonary dysfunction in severe COVID-19.

Neutrophils represent an important source of oxidative injury during severe viral pneumonia. Following activation, neutrophil NADPH oxidase generates superoxide and downstream ROS that participate in antimicrobial defense but can also damage the surrounding pulmonary epithelial and endothelial tissues when produced excessively. In severe COVID-19, neutrophil-derived oxidative stress has been proposed to contribute to alveolar injury, endothelial dysfunction, and the amplification of inflammation. Because GSH is required to detoxify hydrogen peroxide and other reactive intermediates, the depletion of GSH may reduce the capacity of pulmonary cells to contain this neutrophil-associated oxidant burden [35,36].

### 2.4. Reduced Lung Compliance and Its Impact on Pulmonary Gas Exchange

These cumulative effects of oxidative stress, endothelial injury, and microvascular thrombosis within pulmonary tissue directly impair lung mechanics and gas exchange. Pulmonary endothelial damage and microthrombi physically obstruct alveolar capillaries, creating regions of ventilation–perfusion (V/Q) mismatch where alveoli are ventilated but poorly perfused. This mismatch leads to hypoxemia, increases pulmonary vascular resistance, and stiffens the lung parenchyma, reducing static lung compliance and elevating the work of breathing [37]. Postmortem arteriograms of ARDS patients revealed widespread vascular thrombosis with microangiopathy, including alveolar capillary thromboemboli, which was present in 21 out of 22 ARDS patients in the study [38].

Such microvascular obstruction, coupled with inflammation and edema, contributes to the development of ARDS, a hallmark of severe COVID-19. This pathophysiology clinically correlates with refractory hypoxemia and respiratory failure, often necessitating mechanical ventilation. Importantly, GSH depletion exacerbates this cascade by diminishing antioxidant defenses and promoting ROS accumulation. This amplifies endothelial injury and microvascular thrombosis, linking oxidative stress directly to impaired lung function and ultimately to the need for mechanical ventilation in severe COVID-19 patients (Figure 4).

As shown in Figure 4, (1) SARS-CoV-2 binds to ACE2 receptors on alveolar epithelial cells, triggering a local immune response. (2) Infected epithelial cells and macrophages release pro-inflammatory cytokines, which diffuse into nearby pulmonary capillaries and induce the release of procoagulation factors. (3,4) These procoagulation factors promote thrombus formation in the pulmonary microvasculature, reducing blood flow and the perfusion of ventilated alveoli. (5) The decreased perfusion of the capillaries impairs pulmonary gas exchange by limiting carbon dioxide removal and oxygen uptake.

## 3. GSH Depletion Is Associated with Disease Severity and Intubation Status

Karkhanei et al. conducted a case–control study of 96 participants divided into healthy controls, hospitalized non-ICU patients with COVID-19, ICU patients without endotracheal intubation, and ICU patients with endotracheal intubation. Serum samples were obtained within 24 h of admission, and GSH, total oxidant status, and total antioxidant capacity were measured. Mean serum GSH concentrations progressively differed across the four groups: 374.94 ± 41.15 μmol/mL in controls, 227.03 ± 36.91 μmol/mL in non-ICU patients, 134.54 ± 38.11 μmol/mL in ICU patients without intubation, and 102.11 ± 36.86 μmol/mL in ICU patients with intubation (*p* < 0.001). In the opposite direction, mean total oxidant status increased from 1.28 ± 0.75 nmol/mL in controls to 15.40 ± 2.94 nmol/mL in non-ICU patients, 28.13 ± 4.87 nmol/mL in ICU patients without intubation, and 82.89 ± 8.49 nmol/mL in ICU patients with intubation. These cross-sectional findings demonstrate a graded association between greater clinical severity, lower circulating GSH, and higher systemic oxidant burden. Because intubation status was used to define the study groups, however, the findings establish an association with intubation rather than a prospective prediction of the need for mechanical ventilation [39].

The clinical relevance of this redox pattern extended beyond ICU placement and intubation status. Patients with an SpO_2_ below 88% had lower mean GSH concentrations than patients in the higher oxygen saturation categories, and patients hospitalized for more than one week had lower GSH than those hospitalized for one week or less. Mean GSH was also lower among patients who died than among those who survived (113.90 ± 41.31 versus 188.12 ± 62.23 μmol/mL; *p* < 0.001). Conversely, total oxidant status was higher among patients with an SpO_2_ below 88%, those who were hospitalized for more than one week, and those who died. The TOS/GSH ratio also increased across the control, non-ICU, ICU-without-intubation, and ICU-with-intubation groups, indicating that the relationship between oxidant burden and GSH availability separated the clinical groups more strongly than either component considered only in isolation. In mortality discrimination analyses, the reported area under the receiver operating characteristic curve was 0.820 for GSH and 0.907 for TOS. These findings support circulating GSH and related redox measures as markers associated with disease severity and mortality in hospitalized COVID-19 patients. They do not, by themselves, establish that GSH independently predicts intubation, because this study did not report a prospective ventilation prediction model or multivariable adjustment for potential confounders [39].

When interpreted together, the Rahman and Karkhanei findings describe GSH disruption in two distinct but complementary biological compartments. Rahman and MacNee documented the marked depletion of the reduced form of GSH within the pulmonary epithelial lining fluid of patients with ARDS, providing evidence of impaired local antioxidant defense at the alveolar surface [7]. Karkhanei et al. subsequently demonstrated that circulating GSH was lower and systemic oxidant burden was higher across groups with progressively greater COVID-19 severity [39]. These measurements should not be considered interchangeable: bronchoalveolar measurements characterize the local pulmonary environment, whereas serum measurements reflect systemic redox status. Nevertheless, the concordant direction of the findings supports the broader interpretation that severe respiratory disease is accompanied by the disruption of GSH homeostasis both locally and systemically. The novel clinical question arising from this synthesis is not whether low GSH is present in severe disease but whether prospectively measured changes in compartment-specific GSH, GSSG, or oxidant-to-antioxidant ratios add predictive information beyond established clinical variables. This question remains untested by the studies reviewed here and requires prospective validation.

Across ARDS populations, reported shifts toward lower GSH, higher GSSG, and a reduced GSH:GSSG ratio are generally interpreted as reflecting a state of heightened oxidative burden, suggesting a broader pattern of GSH system perturbation that aligns with both systemic inflammatory stress and local alveolar–capillary dysfunction across diverse ARDS etiologies [40]. Another experiment provided clinical evidence that ARDS is accompanied by the systemic disruption of glutathione homeostasis. In 20 patients with ARDS, the total RBC glutathione pool was reduced, with particularly low glutathionylated hemoglobin, alongside the depletion of other antioxidant defenses such as CoQ10 [41]. These findings suggest that ARDS is characterized by impaired redox buffering, providing a clinical correlate to the ROS-driven mechanisms described in experimental models. Within COVID-19 ARDS, investigators evaluated 65 mechanically ventilated patients with moderate-to-severe COVID-19-associated ARDS and 63 mechanically ventilated non-ARDS controls, measuring reduced glutathione (GSH) and oxidized glutathione in bronchoalveolar lavage fluid [42]. ARDS patients demonstrated decreased GSH with increased GSSG, indicating substantial pulmonary redox imbalance and oxidative damage. Importantly, markers of fibroproliferation were significantly elevated in ARDS and correlated inversely with GSH and positively with GSSG, linking impaired pulmonary antioxidant capacity to early extracellular matrix remodeling and profibrotic signaling. These abnormalities were also more pronounced in nonsurvivors and were associated with residual CT abnormalities and impaired lung function at 3-month follow-up.

Importantly, serum GSH reflects systemic redox status, whereas alveolar GSH reflects antioxidant defenses within the pulmonary epithelial lining fluid; these represent distinct biological compartments obtained from blood versus bronchoalveolar sampling and are not directly interchangeable measures of lung-specific oxidative injury. In ARDS and critical illness, serum measurements are more commonly used for clinical correlation and outcome prediction due to feasibility and their integration of whole-body inflammatory and oxidative burden, while alveolar measurements are primarily used in mechanistic studies to characterize local pulmonary biology. Because oxidative stress in critical illness involves both systemic inflammatory activation and localized alveolar injury, the modulation of GSH homeostasis may plausibly influence both circulating inflammatory responses and pulmonary epithelial oxidative stress [43].

### Practical Considerations for GSH as a Clinical Biomarker

The translation of GSH into a clinically useful biomarker will require the standardization of the biological compartment, sampling time, analytical method, and the interpretation of results. Serum or plasma measurements are substantially more practical for routine clinical use than bronchoalveolar measurements, whereas intracellular GSH measurements in peripheral blood mononuclear cells may provide complementary information regarding cellular redox status. Because GSH is readily oxidized during sample collection and processing, pre-analytical handling and assay methodology must also be standardized before values can be compared across studies or applied clinically. In prospective studies, blood should ideally be obtained at a predefined baseline time point, such as hospital or ICU admission and before the initiation of investigational GSH directed therapy, with serial measurements thereafter to determine whether changes in GSH, GSSG, or the GSH:GSSG ratio precede clinical deterioration. Importantly, currently available studies do not establish a validated GSH concentration or GSH:GSSG threshold for predicting endotracheal intubation or invasive mechanical ventilation. Therefore, GSH should presently be considered an investigational risk stratification biomarker rather than a stand-alone trigger for ventilatory intervention. Prospective studies should determine clinically useful thresholds using receiver operating characteristic analysis and evaluate whether GSH-related measures provide predictive value beyond established variables such as the PaO_2_/FiO_2_ ratio, SpO_2_, oxygen requirement, respiratory effort, and overall illness severity.

## 4. Therapeutic Implications and Biomarker-Guided GSH Strategies

Clinical studies have demonstrated a measurable role for GSH in ARDS, particularly through the pharmaceutical modulation of oxidative stress and gas exchange. Randomized and prospective ICU trials have evaluated N-acetylcysteine (NAC) in patients meeting ARDS criteria, many requiring mechanical ventilation. In one randomized controlled trial, ICU patients with ARDS receiving NAC demonstrated significant improvements in objective respiratory parameters, including increases in PaO_2_/FiO_2_ ratios (mean improvement from approximately 120 ± 40 to 160 ± 50 mmHg over the study period), reflecting enhanced oxygenation. These physiologic gains were accompanied by reductions in Sequential Organ Failure Assessment (SOFA) scores and a lower mortality rate in the NAC group (approximately 31% vs. 62% in controls, *p* < 0.05) [44].

Similarly, a prospective clinical trial examined ARDS patients in the ICU and demonstrated that NAC administration increased systemic antioxidant capacity, including elevated plasma thiol concentrations and total antioxidant capacity, reflecting improved GSH availability and redox balance. These biochemical improvements were accompanied by increases in oxygenation indices (PaO_2_/FiO_2_ ratio, mean improvement of ~25–30%), a reduced duration of mechanical ventilation by approximately 2–3 days, and a shorter ICU length of stay compared to standard care. Collectively, these findings support the restoration of GSH-mediated antioxidant defenses as a measurable correlate of improved physiologic and clinical outcomes in ARDS [45].

Within COVID-19 patients experiencing ARDS, intravenous glutathione (IV GSH) was administered to hospitalized patients with active respiratory compromise. A study from India demonstrated that daily IV GSH administration in patients with moderate COVID-19 resulted in significant clinical improvement, using an initial dose of 2400 mg followed by 1200 mg twice daily. IV administration bypasses first-pass metabolism, allowing for higher peak plasma concentrations and more rapid systemic availability [46]. The primary outcome was an improvement of ≥2 points on the WHO 8-point ordinal scale, and secondary outcomes included oxygen independence, progression in disease severity, hospitalization duration, and safety. IV GSH demonstrated relatively rapid clinical benefits, including improved WHO scores within days, reduced oxygen requirements and a decreased likelihood of prolonged hospitalization. These findings suggest that IV GSH may provide relatively rapid clinical benefits in the acute setting.

However, IV GSH is not currently FDA-approved in the United States, limiting its clinical applicability. As a result, oral liposomal glutathione formulations, such as ReadiSorb^®^ Liposomal Glutathione (RLG), have been proposed as adjunctive therapies for respiratory distress. Liposomal encapsulation enhances bioavailability by protecting glutathione from gastrointestinal degradation and facilitating absorption, thereby offering a potential alternative route for GSH repletion.

Supporting this approach, oral liposomal glutathione (L-GSH) supplementation has demonstrated immunomodulatory and antioxidant effects in a randomized, placebo-controlled trial. Cells from individuals with type 2 diabetes exhibited GSH deficiency, elevated oxidative stress markers (malondialdehyde, MDA), and increased pro-inflammatory cytokine IL-6. After three months of L-GSH supplementation, there was a significant reduction in IL-6 levels in both plasma and peripheral blood mononuclear cells compared with placebo, alongside marked reductions in MDA across plasma, PBMCs, and red blood cells. Active, reduced GSH levels were maintained or increased in the L-GSH group, whereas placebo subjects demonstrated declining reduced GSH and rising oxidized GSH [47]. Collectively, these findings demonstrate that liposomal GSH restores redox balance and attenuates systemic inflammatory signaling, particularly IL-6-mediated pathways. Although these results are not specific to ARDS or COVID-19, they provide mechanistic support for the potential role of oral GSH repletion in reducing oxidative stress and inflammatory burden in respiratory disease.

A biomarker-driven therapeutic framework has been proposed to further evaluate the role of glutathione in respiratory distress. In the first phase, the study would mimic the Karkhanei study by determining the validity of the testing of GSH levels within the peripheral blood mononuclear cells of individuals in the ICU with endotracheal intubation (Table 2). Patients at the other end of the spectrum, meaning individuals presenting with respiratory distress, requiring hospitalization but not ICU admission, will also be tested. The results from these groups would be compared to healthy controls and individuals with respiratory distress who do not require hospitalization to determine whether GSH depletion correlates with disease severity (Table 2).

In parallel, inflammatory and thrombotic biomarkers, including IL-6 and D-dimer, would be measured to assess the degree of immunothrombosis [48]. IL-6 is a key pro-inflammatory cytokine and has been shown to predict respiratory failure in COVID-19 patients, with circulating levels elevated in nearly all infectious, traumatic, and inflammatory states, including ARDS [49]. Similarly, D-dimer levels are elevated in both COVID-19 and non-COVID-19 ARDS, and increased admission levels of D-dimer have been associated with higher mortality risk (Figure 5) [50]. Together, IL-6 and D-dimer provide complementary insight into the thrombo-inflammatory state underlying respiratory failure (Table 2).

**Table 2:** Proposed biomarker changes across ARDS severity.

ARDS	GSH (Karkhanei 2021) [39]	SpO2 @ Room Air (Karkhanei 2021) [39]	IL-6 (Herold 2020) [48]	D-Dimer ng/L in ARDS (Bansal 2020) [51]
Mild	181.70 ± 67	≥91	↑ <35 pg/mL	↑ mild elevation
Moderate	177.8	88–90	↑↑	↑↑ elevation predicts worse disease
Severe	106.2	<88	↑↑↑ 80 pg/mL	↑↑↑ Predicts worse disease

This table provides an illustrative summary of reported associations between ARDS severity and selected biomarkers of oxidative stress, inflammation, and coagulopathy. The values and trends presented are derived from individual studies and should not be interpreted as measurements obtained from the same patient population or as a continuous temporal trajectory of ARDS progression. Reported decreases in intracellular GSH levels and peripheral oxygen saturation (SpO_2_) in room air have been associated with greater disease severity, reflecting impaired antioxidant capacity and worsening gas exchange [39]. Similarly, higher levels of systemic inflammatory and thrombotic markers, including IL-6 and D-dimer, have been reported in patients with greater disease severity [48,51]. These findings highlight the interconnected nature of oxidative stress, inflammatory signaling, and immunothrombosis in the pathophysiology of ARDS, supporting the further investigation of biomarkers for disease stratification and potential therapeutic targeting.

Figure 5 proposes and visualizes the temporal changes in key oxidative, inflammatory, and coagulation-related biomarkers throughout the progression of acute ARDS. Early-phase oxidative stress is characterized by a decline in GSH and a rise in ROS, followed by increases in inflammatory cytokines (e.g., IL-6) and coagulation markers (e.g., D-dimer) [47,50,51]. Declining pulmonary function, represented by reduced lung compliance and increasing oxygen requirements, emerges as a later manifestation of progressive alveolar–capillary injury. The model integrates evidence from prior studies to highlight the potential of GSH as an early upstream biomarker of disease progression [47,50].

Once the testing is validated, an adjunct to standard treatment would be supplied in the form of oral ReadiSorb^®^ Liposomal Glutathione (RLG) to evaluate its potential clinical benefit in patients with respiratory distress. Liposomal encapsulation results in rapid absorption and the bypass of the liver’s first-pass metabolism, allowing for rapid GSH restoration. In addition, liposomes have been shown to preferentially accumulate at sites of inflammation, potentially improving targeted antioxidant effects [52].

For future prospective studies evaluating GSH as a predictor or therapeutic target, the primary ventilatory endpoint should be explicitly defined as new initiation of invasive mechanical ventilation through endotracheal intubation after baseline biomarker sampling. Noninvasive respiratory support, including high-flow nasal cannula, continuous positive airway pressure, or bilevel positive airway pressure, should be analyzed separately as escalation-of-support outcomes rather than being included within the primary invasive ventilation endpoint. Secondary respiratory endpoints may include time from baseline sampling to intubation, the maximum level of respiratory support, ventilator-free days, the duration of invasive mechanical ventilation, ICU length of stay, and mortality. In patients already invasively ventilated at enrollment, incident intubation cannot serve as an outcome; therefore, ventilator-free days and the duration of mechanical ventilation should instead be evaluated.

The results of administering RLG to individuals with respiratory distress should be observed to determine whether it reduces the need for endotracheal intubation, slows the progression of respiratory distress, and decreases the duration of mechanical ventilation. In patients already receiving ventilatory support, administration via a nasogastric tube could allow for an assessment of whether GSH repletion shortens the duration of mechanical ventilation compared with standard care alone.

Dosing strategies for oral liposomal glutathione could be designed to approximate previously studied intravenous regimens; however, important pharmacokinetic differences between oral and intravenous administration must be considered. Optimal dosing parameters, including frequency, duration, and total daily dose, remain to be established and should be guided by biomarker response, such as intracellular GSH levels, as well as clinical outcomes. Additionally, safety, tolerability, and variability in absorption may influence dosing requirements, underscoring the need for further investigation in controlled clinical trials.

This proposed framework is applicable across a spectrum of respiratory illness severity, consistent with prior studies evaluating GSH status in patients with varying degrees of respiratory compromise [38]. The clinical outcomes may be standardized by using the WHO ordinal scale for disease severity, as seen in Table 3 [53].

## 5. Methods

A literature search was conducted to identify relevant studies on GSH and its role in inflammation and ARDS ventilation. The search was carried out through electronic databases PubMed, Google Scholar, and ScienceDirect, prioritizing articles published in the last five years. Search terms such as “Glutathione in ARDS”, “COVID-19 ARDS”, and “ARDS mechanical ventilation” were used, with Boolean operators used to combine terms. Filters were applied to include only peer-reviewed articles in English. To ensure comprehensive coverage, the references of relevant articles were manually screened for additional studies.

Studies were included if they met predefined eligibility criteria: (1) they investigated GSH levels, depletion, or supplementation in the context of ARDS or COVID-19-related lung injury; (2) they were conducted in human subjects; and (3) they reported clinically relevant outcomes, such as oxidative stress markers (e.g., ROS, TOS), inflammatory markers (e.g., interleukin-6 [IL-6]), thrombotic markers (e.g., D-dimer), lung function parameters (e.g., PaO_2_/FiO_2_ ratio, oxygen requirements), or clinical outcomes including the need for mechanical ventilation, intensive care unit (ICU) admission, or mortality. Studies investigating related antioxidant strategies, including N-acetylcysteine (NAC), were also considered if their findings were directly relevant to GSH availability, GSH synthesis, or the restoration of cellular antioxidant capacity. Both randomized controlled trials (RCTs) and observational studies were considered eligible, provided they included sufficient quantitative data for review. Studies involving combinations of pharmacological therapies were included only if the effect of GSH could be isolated.

To provide mechanistic context, selected animal and in vitro studies were also included if they addressed biological pathways relevant to GSH depletion, ROS, inflammation, endothelial dysfunction, thrombo-inflammation, or pulmonary injury. These articles, however, were only used to provide mechanistic evidence and were not considered equivalent to clinical evidence in human ARDS.

Exclusion criteria included studies not related to ARDS or COVID-19-associated lung injury, as non-pulmonary conditions differ in pathophysiology and limit applicability to respiratory failure. Studies unrelated to the mechanistic or clinical framework of ARDS and respiratory failure were also excluded unless they provided complementary evidence directly relevant to GSH biology or its relationship to oxidative stress and inflammation. Case reports, editorials, and conference abstracts were omitted to prioritize primary research, while review articles were used selectively for background. Articles lacking measurable outcomes or full-text availability were excluded when necessary.

## 6. Conclusions

Decreased levels of GSH promote a “vicious cycle” characterized by ROS accumulation, endothelial injury, thrombus formation, and ventilation–perfusion mismatch. As oxidative stress worsens, alveolar–capillary integrity declines, leading to impaired gas exchange. Clinically, this contributes to increased oxygen requirements and a greater likelihood of mechanical ventilation in critically ill patients suffering from ARDS and COVID-19. While COVID-19-associated ARDS has provided particularly well-characterized insights into thrombo-inflammation and oxidative injury, these mechanisms reflect broader pathways of endothelial dysfunction and redox imbalance observed across ARDS.

The management of ARDS commonly relies on monitoring oxygenation parameters such as the PaO_2_/FiO_2_ ratio and SpO_2_, which reflect downstream impairment in pulmonary gas exchange and alveolar–capillary function. Declining GSH levels may represent an upstream marker of oxidative stress and cellular injury, identifying patients at risk before changes in oxygenation become clinically apparent. The early identification of GSH depletion could help predict which patients are at the highest risk of ARDS progression [49].

Therapeutic strategies aimed at restoring GSH have demonstrated improvements in surrogate endpoints including oxidative stress markers, inflammatory cytokines, and oxygenation parameters in small or heterogeneous studies. In the oral liposomal GSH study, supplementation restored redox balance, reduced IL-6-mediated inflammatory signaling and preserved reduced GSH status. Intravenous GSH has demonstrated more immediate clinical benefits in patients with acute ARDS, including improved oxygen requirements and reduced disease severity progression.

At present, GSH measurements should not be used independently to determine the timing or initiation of ventilatory support because clinically validated thresholds for predicting invasive mechanical ventilation have not been established. Rather, available evidence supports the further investigation of GSH, GSSG, and related redox indices as candidate biomarkers that may complement established measures of respiratory deterioration, including the PaO_2_/FiO_2_ ratio, SpO_2_, oxygen requirement, respiratory effort, and overall clinical trajectory. Prospective studies with standardized sampling, predefined invasive mechanical ventilation endpoints, and adjustment for relevant clinical confounders are required to determine whether GSH provides independent or incremental predictive value. If validated, serial GSH measurements could ultimately contribute to multimodal risk stratification rather than serve as a stand-alone indication for intubation.

Although larger randomized controlled trials are necessary to confirm clinical efficacy and define optimal timing and dosing strategies, emerging evidence supports GSH repletion as a promising alternative approach in both the prevention and management of inflammatory lung injury. By targeting redox imbalance, GSH repletion may reduce progression to respiratory failure and decrease the need for mechanical ventilation in high-risk patients.

## Figures and Tables

**Figure 1:**
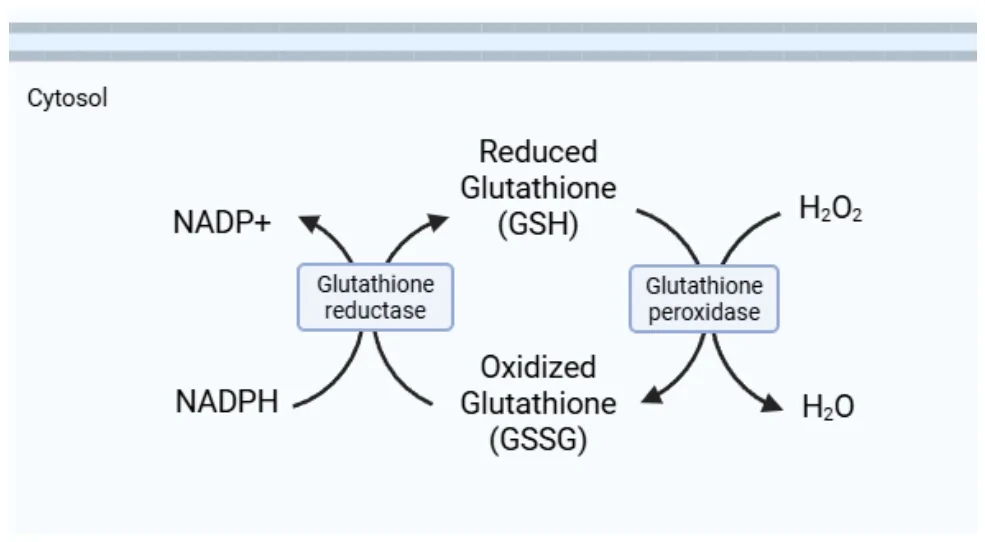
GSH redox cycle.

**Figure 2:**
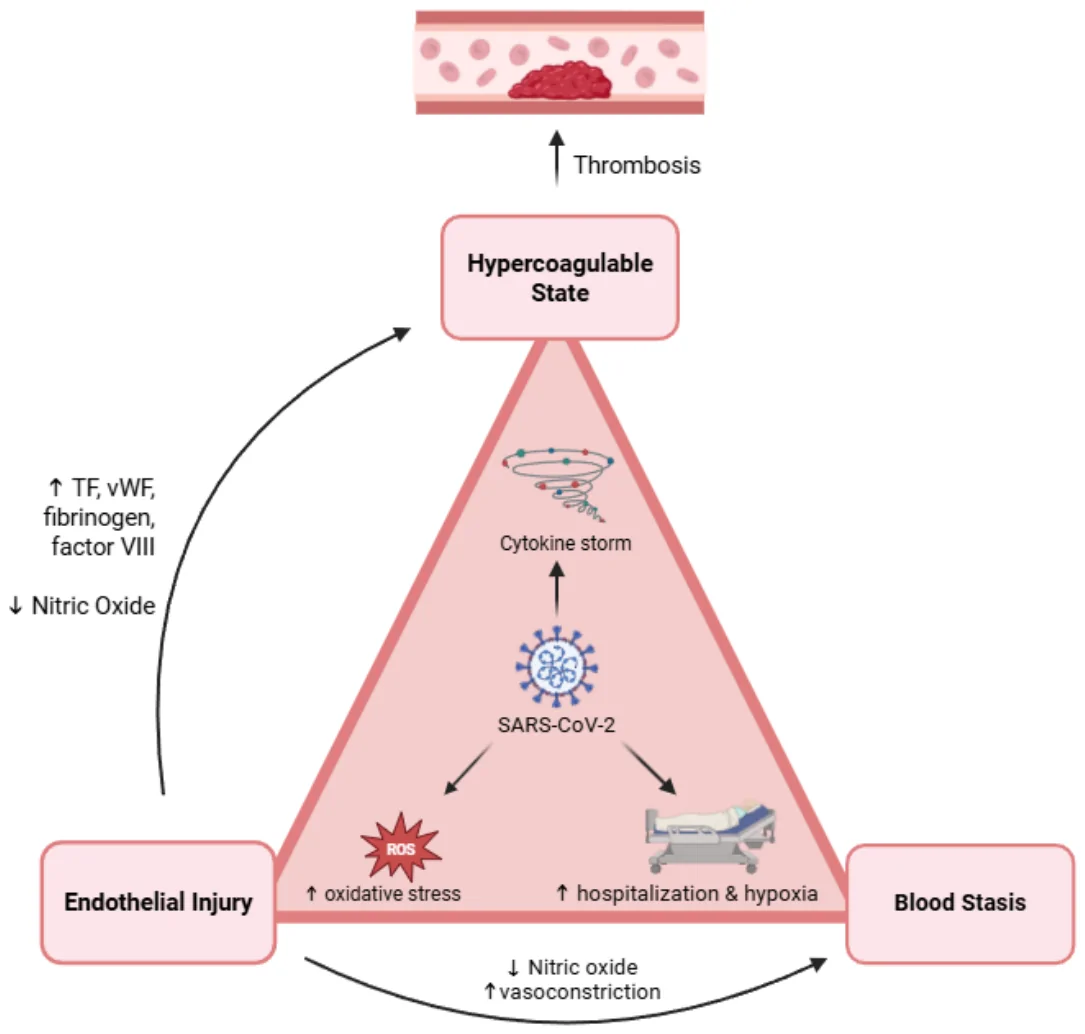
SARS-CoV-2-induced activation of Virchow’s triad.

**Figure 3:**
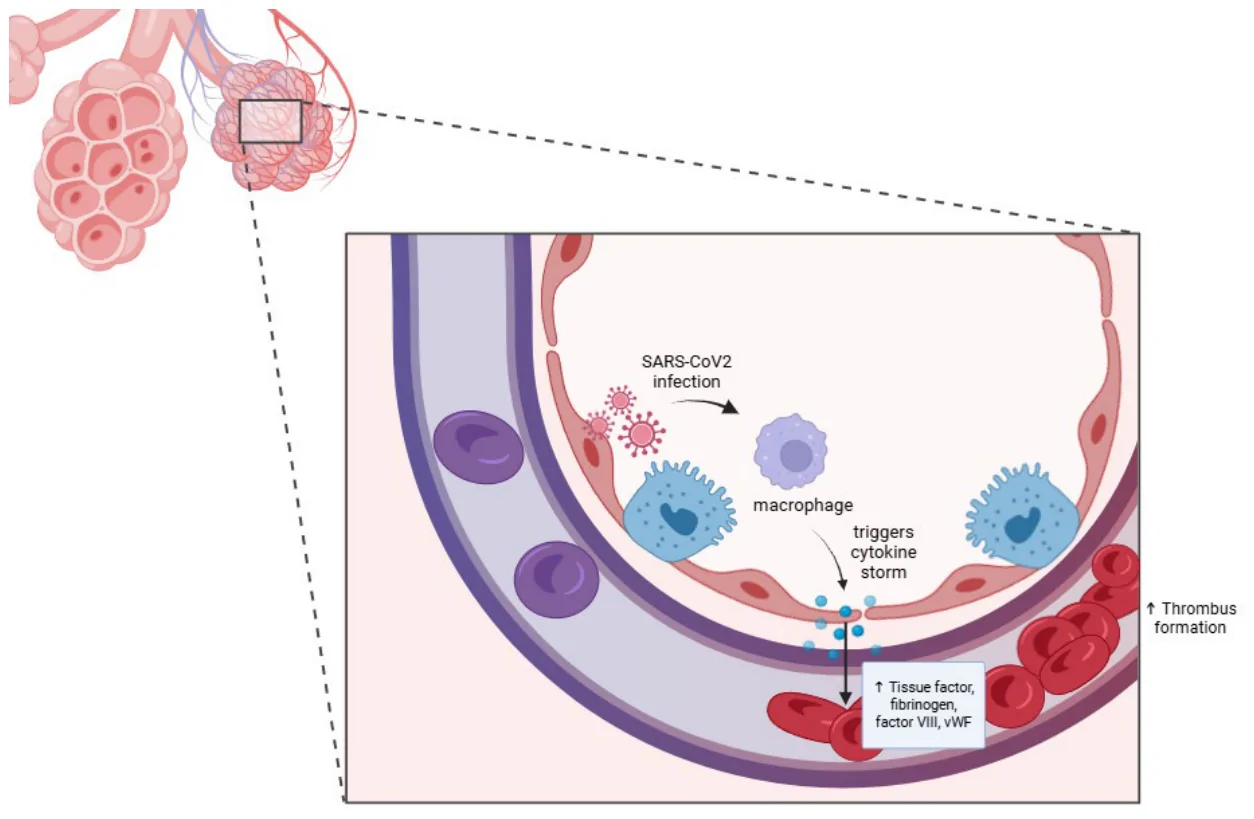
SARS-CoV-2 infection triggers cytokine storm and promotes thrombosis through activation of procoagulation factors.

**Figure 4:**
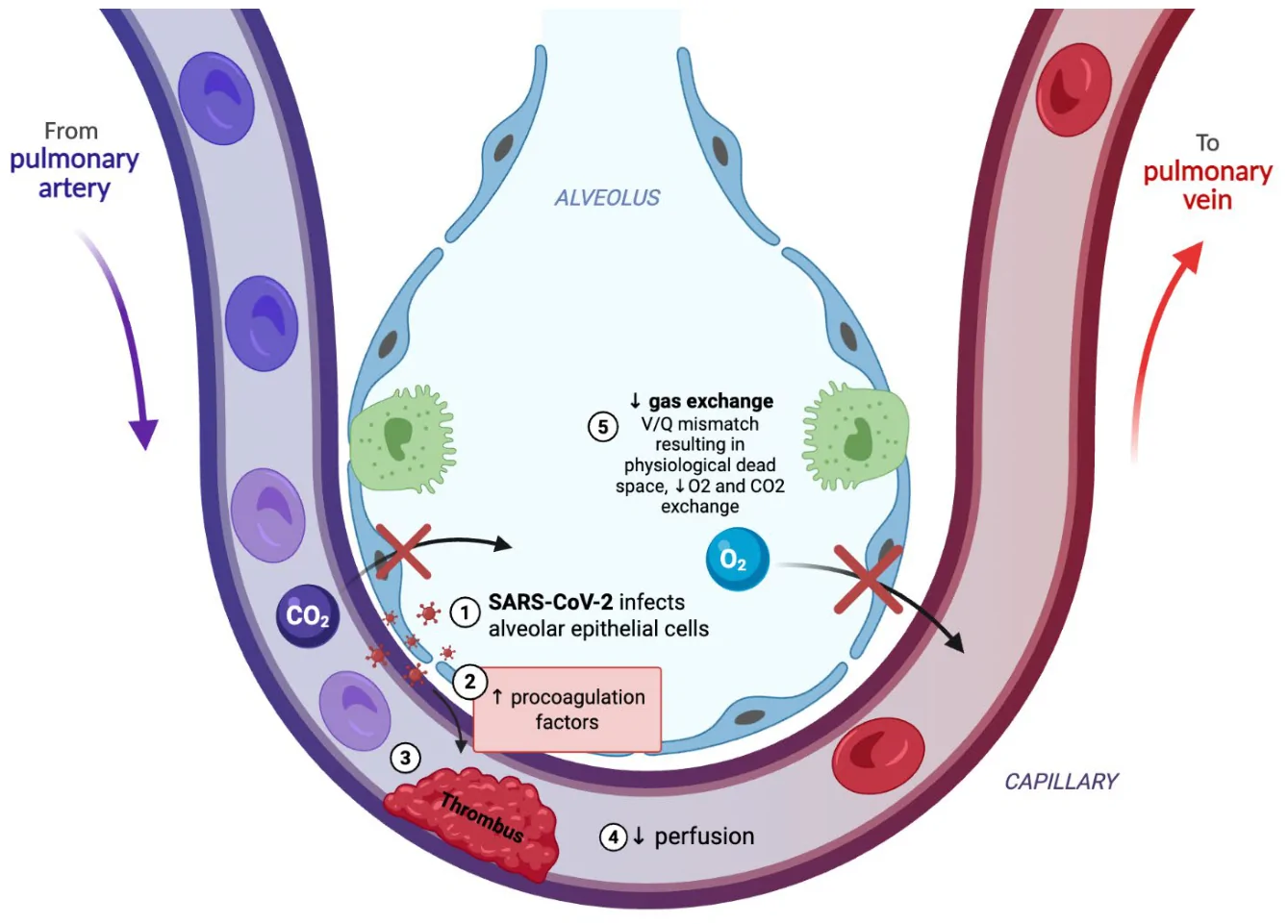
SARS-CoV-2-induced thrombus formation impairs pulmonary gas exchange.

**Figure 5:**
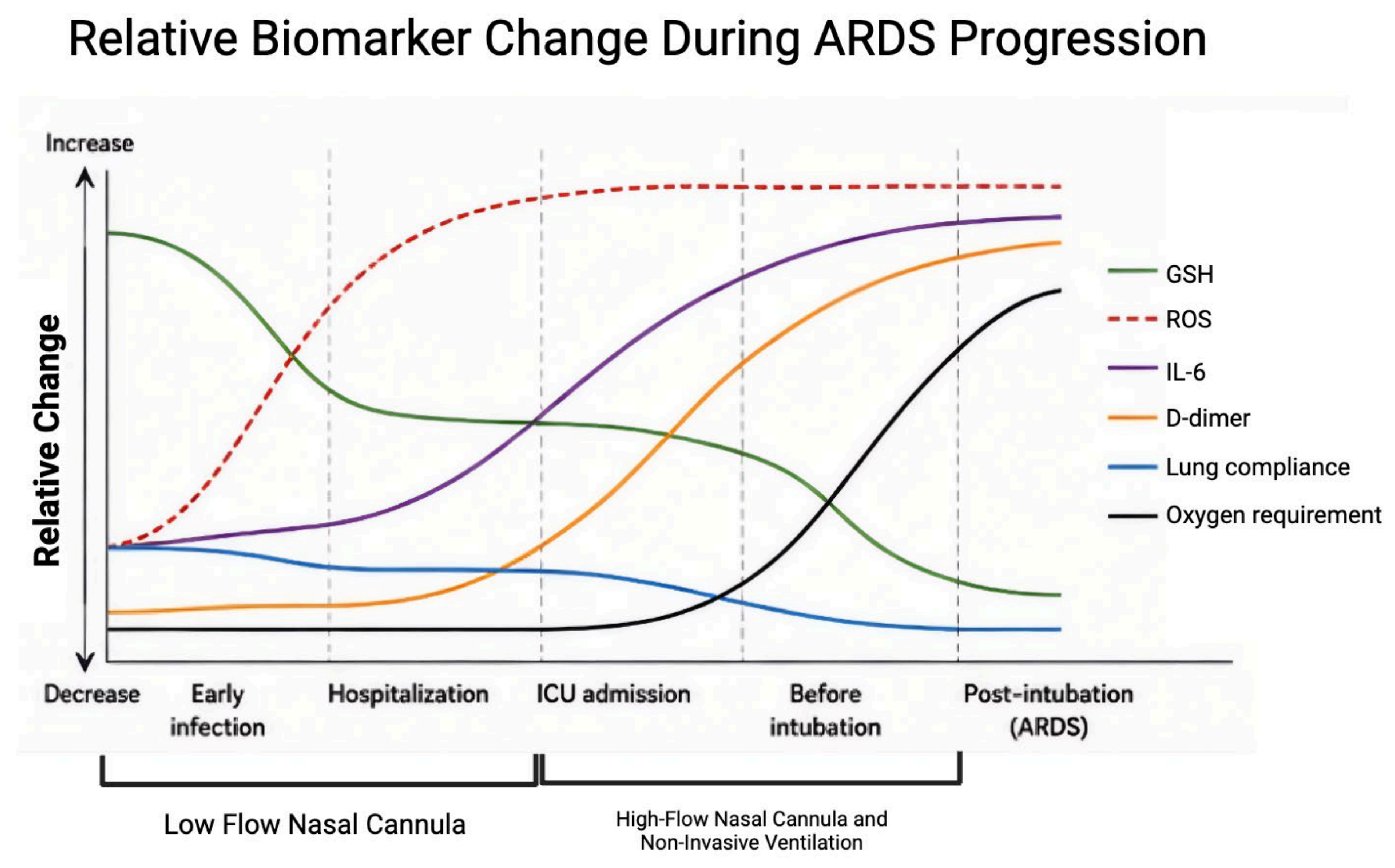
Proposed temporal model of biomarker dynamics during ARDS progression.

**Table 1:** Proposed pathophysiological framework linking glutathione depletion to oxidative stress, endothelial dysfunction, thrombo-inflammation, and progressive respiratory failure in ARDS.

Stage	Key Pathophysiologic Process	Role of Glutathione (GSH)	Representative Biomarkers	Expected Clinical Consequence
Initial insult	SARS-CoV-2 infection, sepsis, trauma, or aspiration initiates pulmonary injury and ROS generation.	Increased ROS consumption lowers intracellular GSH and disturbs redox balance.	↓ GSH, ↑ GSSG	Oxidative stress
Oxidative stress	NOX2 activation and mitochondrial ROS amplify oxidative injury.	Loss of antioxidant buffering permits oxidation of proteins, lipids, and DNA.	↓ GSH/GSSG ratio	Cellular injury
Endothelial dysfunction	Reduced nitric oxide bioavailability and increased vascular permeability.	Reduced GSH may impair endothelial redox homeostasis.	IL-6, ICAM-1, VCAM-1	Vascular leak and endothelial activation
Inflammation	Activation of NF-κB/AP-1 signaling with cytokine production and leukocyte recruitment.	GSH depletion may amplify redox-sensitive inflammatory signaling.	↑ IL-6, TNF-α, IL-1β	Alveolar inflammation
Coagulation activation	Tissue factor expression, platelet activation, fibrin formation, and immunothrombosis.	Oxidative stress is associated with thrombo-inflammatory responses.	D-dimer, fibrinogen, vWF	Microvascular thrombosis
Pulmonary dysfunction	Alveolar–capillary injury and ventilation/perfusion mismatch.	Reduced antioxidant defenses may worsen gas exchange.	↓ PaO_2_/FiO_2_ ratio	Hypoxemia
Advanced ARDS	Progressive respiratory failure with reduced lung compliance.	Severe oxidative stress accompanies advanced disease; predictive value of GSH remains unproven.	↓ Static lung compliance	Mechanical ventilation

**Table 3:** **The WHO ordinal scale of disease severity**. The WHO 8-point ordinal scale is a standardized clinical tool used to quantify disease severity in patients with respiratory illness, particularly in COVID-19 and ARDS. The scale ranges from no clinical or virological evidence of infection to death, with intermediate categories reflecting increasing levels of respiratory support and clinical deterioration. Lower scores indicate ambulatory patients with no or mild activity limitations, while higher scores correspond to hospitalization requiring supplemental oxygen, noninvasive or invasive mechanical ventilation, and advanced organ support such as vasopressors or extracorporeal membrane oxygenation (ECMO). This scale enables the consistent stratification of disease severity and facilitates the comparison of clinical outcomes across studies.

Score	Descriptor
0	No clinical or virological evidence of infection
1	Ambulatory; no activity limitation
2	Ambulatory; activity limitation
3	Hospitalized; no oxygen therapy
4	Hospitalized; oxygen mask or nasal prongs
5	Hospitalized; noninvasive mechanical ventilation (NIMV) or high-flow nasal cannula (HFNC)
6	Hospitalized; intubation and invasive mechanical ventilation (IMV)
7	Hospitalized; IMV + additional support such as pressors or extracardiac membranous oxygenation (ECMO)
8	Death

## Data Availability

No new data were created or analyzed in this study. Data sharing is not applicable to this article.

## Abbreviations

The following abbreviations are used in this manuscript:

ARDS	acute respiratory distress syndrome
AP-1	activator protein-1
GSH	reduced form of glutathione
DVT	deep vein thrombosis
GSSG	oxidized glutathione
ICU	intensive care unit
ICAM-1	intercellular adhesion molecule-1
IL-1β	interleukin-1 beta
IL-6	interleukin-6
NF-κB	nuclear factor kappa B
NOX2	NADPH oxidase 2
ROS	reactive oxygen species
TNF-α	tumor necrosis factor alpha
VCAM-1	vascular cell adhesion molecule-1
vWF	von Willebrand factor;
V/Q	ventilation/perfusion
